# Health related quality of life trajectories and predictors following coronary artery bypass surgery

**DOI:** 10.1186/1477-7525-4-49

**Published:** 2006-08-13

**Authors:** Michael R Le Grande, Peter C Elliott, Barbara M Murphy, Marian UC Worcester, Rosemary O Higgins, Christine S Ernest, Alan J Goble

**Affiliations:** 1 Heart Research Centre Melbourne, Box 2137 Post Office, The Royal Melbourne Hospital, VIC 3050, Australia; 2 The Australian Centre for Posttraumatic Mental Health, The University of Melbourne, Australia; 3 Department of Psychology, The University of Melbourne, Australia

## Abstract

**Background:**

Many studies have demonstrated that health related quality of life (HRQoL) improves, on average, after coronary artery bypass graft surgery (CABGS). However, this average improvement may not be realized for all patients, and it is possible that there are two or more distinctive groups with different, possibly non-linear, trajectories of change over time. Furthermore, little is known about the predictors that are associated with these possible HRQoL trajectories after CABGS.

**Methods:**

182 patients listed for elective CABGS at The Royal Melbourne Hospital completed a postal battery of questionnaires which included the Short-Form-36 (SF-36), Profile of Mood States (POMS) and the Everyday Functioning Questionnaire (EFQ). These data were collected on average a month before surgery, and at two months and six months after surgery. Socio-demographic and medical characteristics prior to surgery, as well as surgical and post-surgical complications and symptoms were also assessed. Growth curve and growth mixture modelling were used to identify trajectories of HRQoL.

**Results:**

For both the physical component summary scale (PCS) and the mental component summary scale (MCS) of the SF-36, two groups of patients with distinct trajectories of HRQoL following surgery could be identified (*improvers *and *non-improvers*). A series of logistic regression analyses identified different predictors of group membership for PCS and MCS trajectories. For the PCS the most significant predictors of *non*-*improver *membership were lower scores on POMS vigor-activity and higher New York Heart Association dyspnoea class; for the MCS the most significant predictors of *non*-*improver *membership were higher scores on POMS depression-dejection and manual occupation.

**Conclusion:**

It is incorrect to assume that HRQoL will improve in a linear fashion for all patients following CABGS. Nor was there support for a single response trajectory. It is important to identify characteristics of each patient, and those post-operative symptoms that could be possible targets for intervention to improve HRQoL outcomes.

## Background

Coronary artery bypass graft surgery (CABGS) is aimed at alleviating patients' morbidity and prolonging their lives. Given the high success rate of such surgery in achieving these aims, it is clear why the assessment of health related quality of life (HRQoL) is of such importance. Longitudinal studies [1-3] have confirmed that most patients report improved HRQoL following surgery through reduced symptoms, improved functioning and increased participation in activities. However, for a significant minority of patients, this improvement does not occur or the patients report a deterioration in HRQoL [4-7]. Thus, potentially there are a number of trajectories a patient may follow after surgery – improve, maintain the same level, deteriorate, or a combination of these. The purpose of the present study was to determine the optimal number of trajectories that best fit the HRQoL data in a sample of CABGS patients. A second aim was to identify patient characteristics that predict these different trajectories.

Most studies that have investigated change over time in CABGS patients [1,3,8-10] have typically used linear models for data analyses, and assumed that individuals follow the same mean trajectory. In repeated measure analysis of variance, the most common approach, no adjustment is made for situations where measurement intervals are unequal. Moreover, traditional methods dismiss individual differences in change as random error [11]. In contrast, growth modelling is a relatively new technique that can be used to estimate parameters and model fit statistics for both linear and non-linear change. Furthermore, modelling packages, such as Mplus [12] have sophisticated routines that permit the inclusion of individuals who were not assessed at all time points. These techniques are increasingly being used to model longitudinal data in repeated measures studies. However, they have only recently been used to examine HRQoL trajectories [11,13].

Wilson and Cleary [14] provide a useful organizing framework for categorising predictors of HRQoL. They distinguish physiological/biological factors, symptoms (including emotional and cognitive variables), individual characteristics, such as gender or age, and environmental characteristics, such as provision of services.

The biological/physiological or medical characteristics that have been consistently associated with poorer HRQoL outcomes after CABGS include pre-surgical cardiac functional status, such as the New York Heart Association (NYHA) classification of dyspnoea [1,15,16], current smoking [1,10], poor left ventricular ejection fraction [1,8], and presence of a comorbidity such as diabetes [9,10,16,17] or pulmonary disease [1,10,17]. Operational variables, such as complications arising from the surgery, may also possibly impact upon HRQoL, but these variables have not been extensively examined [1].

It is not surprising that symptoms of depression or anxiety have been associated with a marked alteration in mental HRQoL and worse outcomes after CABGS. Among patients scheduled for CABGS, the prevalence of depressive symptoms is high, with a recent Australian study finding that over half of bypass candidates were diagnosed as depressed [18]. Preoperative anxiety and depression often predict the occurrence of symptoms or psychopathology after surgery [4,19,20]. Hofer and colleagues [21] provide evidence that HRQoL and depression are distinctive psychological entities, but that depression represents the most important indirect influence on the course of HRQoL in coronary artery disease patients.

Decreased cognitive function has been recognized to be a major, although probably partly reversible, unintended outcome of CABGS [3]. A growing number of studies have investigated the relationship between neurocognitive functioning and HRQoL in CABGS patients, and reported mixed findings. Studies using a composite cognitive index have found strong associations between neurocognitive functioning and change in HRQoL following CABGS [22,23]. In contrast to these findings, a recent longitudinal study [3] found no significant association between HRQoL and cognitive performance. Further investigation of the association between cognitive functioning and HRQoL is warranted.

Among the individual characteristics that have been associated with poorer HRQoL outcomes after coronary interventions are age and gender. Younger patients [24,25] have reported lower mental HRQoL scores, and older patients have reported lower physical HRQoL scores [1,10,24]. There have been mixed findings with gender, but studies that have controlled for age and other relevant variables have reported lower HRQoL in females [10,17,25-27]. Other variables such as living alone [28], unemployment [26] and lower socio-economic status [29] have been associated with poorer mental and physical HRQoL.

One possible environmental influence on HRQoL outcomes is cardiac rehabilitation (CR) program attendance. Despite its efforts to improve the psychological, as well as the physical, well-being of patients [30], CR attendance has produced inconsistent associations with HRQoL outcomes. A number of studies have found CR attenders had no better HRQoL than non-attenders at follow up [31,32], while other studies have found more positive effects [33].

The aims of the present study were:

a. to identify the general form of change of HRQoL over time, i.e. linear or non-linear, using growth curve modelling (GCM)

b. to identify the different trajectories of HRQoL over a six month period for both physical health and mental health, using growth mixture modelling (GMM)

c. to identify the socio-demographic, medical, psychological or cognitive variables that predict group membership of HRQoL trajectories.

## Methods

### Patients

Eligible patients were adults on the waiting list for CABGS at The Royal Melbourne Hospital, Australia, between July 2001 and April 2004. Patients were excluded from the study if they were under the age of 18 or over 85 years of age; were subsequently assigned to a non-CABGS procedure; or refused consent. Patients could withdraw from the study at any stage between consent to participate and the final follow-up assessment at six months. The following were criteria for withdrawal: failure to return postal questionnaires, death, physical illness or frailty preventing participation, occurrence of a major medical or neurological illness that would independently affect cognitive outcome, refusal, and unavailability for follow-up for other reasons.

Of a consecutive sample of 444 patients to whom a questionnaire package was posted, 220 (49%) returned the questionnaires. The mean interval between completion of the baseline questionnaire and surgery was 33 days (SD = 34 days; median = 26 days). Of the returned questionnaires, 37 were excluded because, based on medical records, it was ascertained that the patient had not undergone CABGS. In order to ascertain reasons for non-completion or non-return of questionnaires, a random sample of one in three patients (n = 78) was contacted by telephone. Amongst these patients, reasons for non-completion of the questionnaire were language difficulties (42%), refusal (38%), death (12%) or disability (8%). The 261 patients who were either excluded or did not return questionnaires were compared with the 183 included patients on all medical variables, gender and age. Excluded patients were less likely to have either high cholesterol (χ^2 ^= 10.6, df = 1, *p *=.001) or a positive family history of cardiovascular disease (χ^2 ^= 4.6, df = 1, *p *= .033). There were no significant differences in all other medical variables, gender and age. Of the 183 returned questionnaires, HRQoL data were available for 182 patients.

To determine whether there was any systematic bias to the analyses due to participant dropout over the six months, the socio-demographic and pre-operative medical characteristics of patients who completed the HRQoL measure at all three time points (*n *= 117) were compared with those of the patients who did not complete it at any of these time points (*n *= 65). There were no significant differences between dropouts and completers on any pre-operative medical or socio-demographic characteristics.

### Data collection

Institutional ethics committee approval was obtained for this study. Names and addresses of patients were obtained from the list of patients waiting for CABGS at The Royal Melbourne Hospital, Australia. Patients were posted the questionnaire package, which included a covering letter, signed by the Head of the Cardiothoracic Surgery Unit, outlining the study and requesting patient consent to participate. Questionnaires were completed prior to surgery, and again at two and six months after CABGS. All questionnaires were returned by reply-paid post. To reduce response bias, the order of presentation of the instruments within the questionnaire was systematically varied across the study population.

## Measures

### Socio-demographic variables

Socio-demographic data collected included gender, age, country of birth, main language spoken, marital status, number of people in the household, school leaving age, highest level of education, current employment status, and current or last occupation. Occupations were classified into nine categories using the Australian Standard Classification of Occupations [34] and then further grouped as manual occupations or non-manual occupations using categories devised by the Australian Institute of Health and Welfare [35].

### HRQoL

The (Short-Form) SF-36 [36] was used to measure perceptions of health outcomes. The physical component summary (PCS) and the mental component summary (MCS) score reflect, respectively, a patient's overall physical and mental health status [37]. The PCS and MCS scores have a possible range of 0 to 100, where higher scores indicate better health status. The summary scores are standardized to the general population (M = 50; SD = 10). Very high PCS scores indicate no physical limitations, disabilities or decrements in well-being as well as a high energy level. Very low PCS scores indicate substantial limitations in self-care, physical, social and role activities; severe bodily pain; or frequent tiredness. Very high MCS scores indicate frequent positive affect, absence of both psychological distress and limitations in usual social/role activities due to emotional problems, while very low MCS scores indicate frequent psychological distress and substantial social and role disability due to emotional problems. A comprehensive literature review [38] of the psychometric properties of instruments used to measure HRQoL among people with heart disease concluded that the most appropriate generic measure for cardiac patients was the SF-36. Chronbach alpha reliability coefficients calculated in the present study were .89 for the PCS and .87 for the MCS. Scoring and interpretation of the SF-36 followed the methods described in the SF-36 manual [39].

### Mood

Patient mood was assessed by the Profile of Mood States (POMS) [40]. The POMS consists of 65 five-point adjective rating scales that identify six moods or affective states: fatigue-inertia, depression-dejection, vigor-activity, anger-hostility, tension-anxiety and confusion-bewilderment. Respondents rate the adjectives on a 5-point intensity scale, in terms of how they have been feeling in the past week (0 = not at all to 4 = extremely). Except for vigor-activity, the higher the score, the greater the mood disturbance/more distress. The reliability and validity of the POMS has been well established for use with cardiac surgical patients [41,42]. In the present study Chronbach alpha coefficients were .91 for tension/anxiety, .94 for depression-dejection, .89 for anger/hostility, .85 for vigor-activity, .91 for fatigue-inertia and .80 for confusion-bewilderment.

### Cognitive functioning

A modified 28-item version of the Everyday Functioning Questionnaire (EFQ) [43] was used to assess patient perceptions of their difficulties with everyday functioning. The EFQ is divided into four sections, with items addressing difficulties in the areas of concentration, memory, communication and organisation. Patients rate all of the questions on a 10 cm visual analogue scale, with possible responses from "no problem" to a "big problem." For all sections, the higher the score the greater the perceived difficulties in that particular component of cognition. Chronbach alpha reliability coefficients were .92 for memory, .89 for concentration, .83 for organisation and .92 for communication.

### Medical variables

A range of pre-surgical, surgical and post-surgical variables was obtained from hospital medical records. Many of these variables were identified in the published literature as risk variables for mortality and health outcomes after CABGS [1,4,8-10,44]. These variables included past surgical history, previous myocardial infarction (MI), presence of co-morbidities, smoking status, body mass index (BMI) and NYHA dyspnoea grade [45]. Surgical data included left ventricular ejection fraction, cross clamp time, pulmonary pressure, number of distal anastamoses and number of diseased vessels. Post-surgical complications such as cardiac arrhythmias, stroke and infections were also recorded. Attendance at a CR program, including number of sessions attended, was determined by contacting the relevant program coordinators.

### Data analysis

For each SF-36 summary scale, modelling was carried out in two stages. At Stage 1 GCM was undertaken. With GCM, individuals are assumed to come from one population and a mean growth curve can be estimated using all individuals. The latent variables representing the intercept (i.e. estimated baseline scores) and trajectory (i.e. estimated change over time) for each sub-scale were derived from the sub-scale scores obtained at the three time points.

Three separate trajectory shapes were tested: linear, square root and quadratic. While a linear function assumes constant change, a square root function assumes more rapid change in the early months, and a quadratic function assumes more rapid change in the later months. These functions were selected from a larger number of possible functions because they represented the most likely trajectory shapes. Selection of the best trajectory shape for each summary scale was based on two commonly used measures of model fit: χ^2 ^value and Comparative Fit Index (CFI) [46]. Lower χ^2 ^values and higher CFI scores are indicative of a better fit of the model to the data. More specifically, CFI values greater than .900 indicate a good model fit [46].

In order to examine the extent to which the intercept and slope were related to the medical socio-demographic and emotional variables the two model parameters were regressed on the 16 potential predictors. A significant coefficient for the term "Intercept regressed on predictor" indicates that the baseline (Physical or Mental) HRQoL varies according to the level of the predictor. A significant coefficient for the term "Slope regressed on predictor" indicates that the change over time in HRQoL varies according to the levels of the predictor.

The second stage of analysis involved GMM. Unlike GCM, GMM calculates fit statistics for sub-groups of individuals called "mixtures", each described by a different growth curve. Hence, GMM utilises more fully the heterogeneity that results from variation in sub-scale scores over time [47]. Lower Bayesian Information Criterion (BIC) values are indicative of a better model fit [48]. The Mplus statistical software [12] was used for all GCM and GMM analyses.

In order to determine the predictors of PCS and MCS sub-groups, a screening process and a series of logistic regression analyses were undertaken. The screening process aimed to optimize the number of potential predictors to be included in the multivariate analysis in order to reach a satisfactory compromise between making Type I and Type II errors. Two screening strategies were adopted. First, dichotomous variables were excluded from further consideration if there were fewer than 10 cases endorsed in any of the two response categories. Second, Chi-square and one-way analysis of variance were used to explore differences between identified sub-groups for variables measured using nominal and interval scales, respectively. Removing those variables that had p > .10 resulted in a reduction of pool predictors from 48 to 12 for PCS and from 48 to 17 for MCS, thereby reducing the probability for type I errors. Using a p-value of > .10 rather than >.05 reduced the likelihood of type II errors.

The remaining independent variables associated with the outcome variables with a p-value of = .10 were then included in a series of logistic regression analyses. For each SF-36 summary scale logistic regression analyses were run separately for socio-demographic variables, medical variables, psychosocial scores and cognitive symptoms. The significant predictors from these analyses were included in two final logistic regression analyses; one for PCS and one for MCS. A backwards regression approach (p < .05 to remain in model) was used for all logistic regression analyses.

Descriptive statistics including mean, standard deviations, frequencies and percentages were used to summarize and present the data. These analyses were performed using SPSS version 13.0 [49].

## Results

### Characteristics of the sample

Pre-operative characteristics of the study population are detailed in Table 1. The average age was 65 years and more than three-quarters of the sample was male. The sample had a high level of comorbidity and a mean BMI in the overweight classification. Almost one-third had NYHA class III or IV, indicative of marked limitations in physical activity. Almost half of the sample had a previous MI and around 10% had previous cardiac surgery.

**Table 1:** Baseline characteristics of the study population

*Demographic*	Mean	(SD)	%
Age	65.5	(9.8)	
Male sex			80
Currently in workforce			33
Currently has partner			74
Non-manual occupation			52
Years education	10.9	(2.5)	
English main language spoken			85
Australian born			60
Lives with others			82
*Cardiovascular disease risk factors*			
Hypertension			86
Diabetes mellitus			29
Cerebrovascular disease			14
Peripheral vascular disease			14
Chronic obstructive pulmonary disease			11
Past smoker			32
Current smoker			10
Body mass index	28.9	(4.6)	
*Cardiac*			
Previous cardiac surgery			11
Previous myocardial infarction			48
Family history of coronary artery disease			40
New York Heart Association functional class			
I			28
II			42
III			28
IV			2
Reduced ejection fraction (EF < .55)			47
*Operational/Post-operational*			
New cardiac arrhythmia			29
Red blood cell transfusion post-op			22
Pulmonary blood pressure	19.3	(5.1)	
Number of distal anastomoses	2.9	(1.0)	
Post-operative ventilation hours	8.3	(6.7)	
Total time in intensive care (hours)	33.9	(18.8)	
Length of stay in hospital (days)	7.1	(5.2)	
Readmitted to hospital within 30 days discharge			12
Attended cardiac rehabilitation program			73
Number of cardiac rehabilitation sessions attended	4.6	(3.4)	
*Health related quality of life (SF-36)*			
Baseline physical component summary	36.6	(10.5)	
Two month physical component summary^a^	39.2	(8.1)	
Six month physical component summary^b^	45.4	(10.4)	
Baseline mental component summary	46.8	(11.2)	
Two month mental component summary^a^	49.7	(11.0)	
Six month mental component summary^b^	50.6	(10.8)	

*n *= 182

a *n *= 128

b *n *= 114

### Growth curve modelling of SF-36 summary scales

Table 2 reports the growth curve model fit statistics and parameter estimates for the Stage 1 analyses. Given the ability of Mplus to use all available data, the sample sizes shown include all patients with component summary scores on at least one occasion.

**Table 2:** Fit statistics and parameter estimates for growth curve models

Component	Form	χ^2^	*p*	CFI	Intercept	*SE*	Slope	*SE*
*Physical*	Linear	4.29	.115	0.963	36.43*	0.69	1.37*	0.16
	Quadratic	7.77	.020	0.908	37.46*	0.65	0.21*	0.03
	Square root	14.02	.001	0.808	35.92*	0.76	3.04*	0.41

*Mental*	Linear	5.31	.07	0.966	47.27*	0.78	0.61*	0.16
	Quadratic	8.92	.01	0.928	47.69*	0.77	0.08*	0.03
	Square root	3.05	.22	0.989	46.90*	0.81	1.61*	0.39

n = 182

For χ^2 ^values df = 2.

CFI = Comparative Fit Index. A higher CFI indicates better model fit.

* *p *< .001

For the PCS, a linear trajectory was found to provide the best fit of the data. Combined with a positive slope parameter, this suggests that PCS scores improved at a steady rate (1.37 units per month) over the six months. For the MCS, a square root function provided the best model fit. Again, combined with the positive slope parameter this indicates that MCS scores improved more rapidly in the first two months followed by relatively less improvement between two and six months. As shown in Table 2, the linear and square root models for the two summary scales yielded non-significant χ^2 ^values and CFIs well above the acceptable level of .900, indicating very good model fit. For both sub-scales the intercept and slope parameters were highly significant.

Table 3 shows the results of regressing the intercept and slope on the potential predictors. Note that a negative coefficient for the Intercept regressed on predictor term indicates that the baseline (Physical or Mental) HRQoL is lower for higher scores on the predictor. A negative correlation for the slope regressed on predictor indicates slower improvement in HRQoL over time for higher scores on the predictor.

**Table 3:** Coefficients for Intercept and Slope Regressed on Predictors

Predictor	Intercept regressed on predictor		Slope regressed on predictor	
	Coeff.	Std. Err.	Coeff.	Std. Err.

*Physical Component Summary*				
NYHA functional class	-2.15*	0.87	-0.13	0.21
Peripheral vascular disease	-4.99*	2.01	-0.24	0.53
POMS tension-anxiety	-0.23*	0.09	0.01	0.02
POMS depression-dejection	-0.13*	0.06	0.01	0.02
POMS vigor-activity	0.39***	0.10	-0.02	0.03
POMS fatigue-inertia	-0.52***	0.09	0.04	0.02
POMS confusion-bewilderment	-0.32*	0.15	0.05	0.04

*Mental Component Summary*				
Partner status	-1.40	1.86	-2.07*	0.90
Previous cardiac surgery	-5.79*	2.57	-1.31	1.26
POMS tension-anxiety	-0.92***	0.09	0.13**	0.05
POMS depression-dejection	-0.64***	0.05	0.07*	0.04
POMS anger-hostility	-0.74***	0.08	0.13**	0.05
POMS vigor-activity	0.69***	0.11	-0.03	0.06
POMS fatigue-inertia	-0.89***	0.10	0.13*	0.05
POMS confusion-bewilderment	-1.37***	0.15	0.16*	0.09

* p < 0.05, ** p < 0.01, *** p < 0.001

For the PCS, lower baseline HRQoL was associated with poorer NYHA functional class; peripheral vascular disease; higher POMS tension-anxiety, depression-dejection, fatigue-inertia, confusion-bewilderment; and lower vigor-activity. No variables were significantly associated with change in physical HRQoL over time.

For the MCS, lower baseline HRQoL was associated with previous cardiac surgery; higher POMS tension-anxiety, depression-dejection, anger-hostility, fatigue-inertia, confusion-bewilderment; and lower vigor-activity. Greater improvement in mental HRQoL over time was associated with having a partner and higher scores on POMS tension-anxiety, depression-dejection, anger-hostility, and fatigue-inertia.

### Growth mixture modelling of SF-36 summary scales

Table 4 provides the parameter estimates and model fit statistics for the growth mixture models. For the PCS a two-group model yielded the lowest BIC and therefore a better fit than the one-group or three-group solutions. The two-group model was also a better outcome than the three-group model because it had a more even distribution of numbers in each group, while still retaining a substantial number in the smallest group. A two-group solution was also the best fitting model for the MCS sub-scale and provided approximately equal numbers in each sub-group.

**Table 4:** Fit statistics and parameters for growth mixture models

				Intercept		Slope	
Model	Group	*N*	BIC	Parameter	*SE*	Parameter	*SE*
*Physical*							
1 Group	1	182	3074.35	36.43*	0.66	1.372*	0.16
							
2 Group	1	108	3073.06	38.63*	1.03	2.158*	0.25
	2	74		33.48*	1.20	0.229	0.25
							
3 Group	1	1	3080.88	47.54*	0.98	-4.683*	0.25
	2	77		33.43*	1.07	0.342	0.21
	3	104		38.66*	1.04	2.223*	0.183

*Mental*							
1 Group	1	182	3152.53	46.87*	0.82	1.595*	0.40
							
2 Group	1	94	3122.05	49.22*	1.55	3.537*	0.61
	2	88		44.60*	1.63	-0.282	0.85
							
3 Group	1	86	3154.28	44.00*	1.90	0.511	0.92
	2	3		53.48*	1.91	-10.798*	1.28
	3	93		49.42*	1.59	3.550*	0.63

Note: BIC = Bayesian Information Criterion. A lower BIC indicates better fit.

* *p *< 0.00

**Figure 1:**
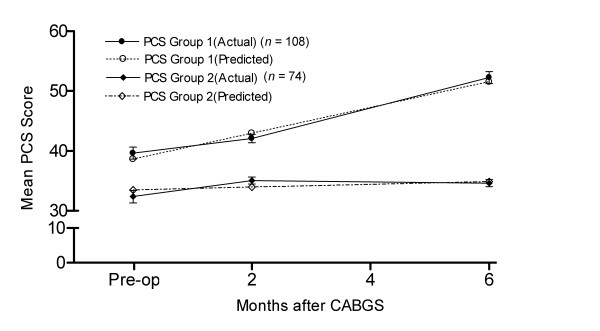
**Predicted and actual trajectories for the PCS**. Note: error bars represent standard error of the mean.

Figure 1 displays both predicted and actual group trajectories for the two-group model for the PCS. The heavier lines represent the actual scores. The divergence of the predicted lines from the actual lines is very small, thus reflecting the good fit of the model to the data. Individuals in group 1, representing 59% of patients, improved rapidly in the first two months after surgery and continued to improve to six months. In contrast, group 2 had a relatively low PCS score before surgery than group 1, and did not change significantly over time.

**Figure 2:**
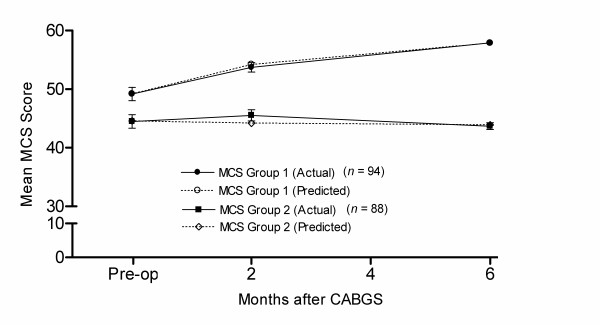
**Predicted and actual trajectories for the MCS**. Note: error bars represent standard error of the mean.

The predicted and actual group trajectories for the two-group model for the MCS are presented in figure 2. As with the PCS, the lack of divergence between actual and predicted scores reflects the good fit of the model to the data. The trajectory patterns are also very similar to the PCS model. Group 1, which represents 52% of participants, improved rapidly in the first two months after CABGS and continued to improve to six months. Patients in group 2 had a lower pre-operative MCS score and, again, showed no significant change over time.

### Predictors of non-improver membership

#### Bivariate screening predicting PCS status

The screening process found that three categories of variables were significant bivariate predictors of PCS group status. For the medical variables, a greater likelihood of being in the *non-improver *group was the presence of atrial fibrillation (*p *< .10), having chronic heart failure (*p *< .05), higher BMI (*p *< .10), having a previous MI (*p *< .05), higher NYHA functional status (*p *< .001), having cardiac arrhythmia after surgery (*p *< .05), and higher pulmonary pressure recorded during surgery (*p *< .05). For the socio-demographic variables *non-improver *status was associated with being female (*p *< .10), being born outside of Australia (*p *< .05), and not being in the workforce (*p *< .05). For the psychosocial variable only lower scores on POMS vigor-activity (*p *< .001) and higher scores on POMS fatigue-inertia (*p *< .05) were associated with *non-improver *status. No cognitive variables distinguished *improvers *from *non-improvers *for the PCS.

#### Logistic regression analysis predicting PCS status

When the seven medical variables were included in a logistic regression to predict PCS group, three (higher NYHA dyspnoea class, new cardiac arrhythmia, and higher mean pulmonary pressure) were significant unique predictors of *non-improver *membership. Of the three socio-demographic variables, being not in the workforce was a significant unique predictor of non-improvement. From the psychosocial variables, both POMS (lower) vigor-activity and (higher) fatigue-inertia, remained significant predictors in the third multivariate analysis.

Table 5 presents the results of the logistic regression analyses using all variable types to predict group membership. For the PCS, factors that were significantly associated with a greater likelihood of being a member of group 2 (*non-improvers*) were: non-participation in the workforce, lower POMS vigor-activity scores, higher NYHA dyspnoea class, experiencing a new cardiac arrhythmia during or following the operation, and higher pulmonary pressure recorded during the procedure

**Table 5:** Factors associated with *non-improver *categories of PCS and MCS trajectories

Component summary	Odds ratio	Lower 95% CI	Upper 95% CI	*p*
*Physical*				
POMS^a ^vigor-activity	0.93	0.87	0.97	.003
NYHA^b ^functional class	1.87	1.18	2.95	.007
New cardiac arrhythmia	2.62	1.13	6.05	.024
Pulmonary pressure	1.09	1.01	1.17	.030
Not in workforce	2.38	1.02	5.56	.045

*Mental*				
POMS^a^depression-dejection	1.08	1.03	1.13	.002
Manual occupation	2.66	1.25	5.65	.011
EFQ^c ^concentration	1.30	1.02	1.66	.037
Previous cardiac surgery	5.17	1.09	27.74	.044

a Profile of Mood States

b New York Heart Association

c Everyday Functioning Questionnaire

#### Bivariate screening predicting MCS status

The screening process found that there were significant bivariate predictors of MCS group status from all four categories of variables. For the medical variables, a greater likelihood of being in the *non-improver *group was the presence of atrial fibrillation (*p *< .10), having off-pump surgery (*p *< .10), having had previous cardiac surgery (*p *< .001), having a previous MI (*p *< .10), and non-attendance at CR (*p *< .05). For the socio-demographic variables *non-improver *status was predicted by not having a current partner (*p *< .10) and having a manual occupation (*p *< .05). For the psychosocial variables *non-improver *status was associated with POMS tension-anxiety anger-hostility, vigor-activity, fatigue-inertia, confusion-bewilderment and depression-dejection (all *p *< .001). For the cognitive variables *non-improver *status was associated with higher EFQ memory (*p *< .05), organisation (*p *< .001), communication (*p *< .05), and concentration (*p *< .001).

#### Logistic regression analysis predicting MCS status

The four separate logistic regressions identified the following variables as significant unique predictors of non-improvement: previous cardiac surgery, previous MI, lower CR attendance, manual occupation, higher (poorer) scores on POMS depression-dejection, and higher (poorer) scores on EFQ concentration.

Table 5 presents the results of the logistic regression analyses using all variable types to predict group membership. For the MCS, factors significantly associated with increased probability for membership of group 2 (*non-improvers*) included: previous cardiac surgery, manual occupation, higher scores on POMS depression-dejection and higher scores on EFQ concentration.

## Discussion

The present study supports previous findings [1-3,7,50] that, on average, HRQoL improves over time following CABGS. However, this study has gone further than others by demonstrating that this improvement is not necessarily linear or applicable to all patients. The overall linear improvement of the PCS to six months conforms with traditional expectations of recovery following surgery. From a physical viewpoint it appears that, overall, patients experience a steady and consistent abatement of their physical symptoms and resumption of activities in the six months following surgery. In contrast, the overall trajectory of patients on the MCS fits a square-root curve rather than the expected linear function. It appears that patients tend to experience a more rapid improvement in emotional status in the early weeks following surgery, but that their return to normal emotional roles and social functioning is much slower than physical functioning in the subsequent months. This relatively slow return to normality for mental functioning, compared with physical functioning, is consistent with findings of other studies [3,7,29].

For the total sample there were significant medical and psychosocial predictors of baseline physical HRQoL including poorer NYHA functional class, having peripheral vascular disease and poorer emotional state. Baseline mental HRQoL was predicted by only previous cardiac surgery among the medical variables and poorer scores on all emotional scales. No cognitive variables significantly predicted baseline HRQoL. For the total sample there were no significant predictors of change in physical HRQoL over time. The apparent contradictory findings that poorer emotional scores predicted greater improvement over time in mental HRQoL may be explained by the fact that these patients had much lower scores at baseline and had greater capacity to change over time. It does appear that having a partner was beneficial for recovery in mental HRQoL and this finding is consistent with previous studies [26,51].

It is important to note that these results only relate to the trajectory of the whole sample. These overall trends, may mask the true picture of recovery following CABGS. The findings of the present study support the hypothesis that identifiable sub-groups of patients exists, each described by a different growth curve, and that these different groups may have different outcomes. Indeed, in this sample, there are two distinct groups of patients for both the physical and mental components of HRQoL. The GMM has identified a larger proportion of the patient population who experience rapid and continued improvement in PCS and MCS scores over time. Of serious clinical relevance, however, the analysis has also identified a smaller group of patients who experience little or no improvement during the first six months following surgery.

The findings of this study lend support to investigation of a range of biopsychosocial predictors, such as that advocated by Wilson and Cleary [14], in explaining the different trajectories patients follow after CABGS. A physiological variable, previous cardiac surgery, was found to be a strong predictor of *non-improver *membership for the MCS. Characteristics of the individual, namely not being in the workforce and having a manual occupation, were predictive of *non-improver *membership for the PCS and the MCS respectively. These findings are consistent with those of past studies [26,52]. It is known that those patients with a manual occupation tend to have a lower socio-economic status, have poorer dietary and exercise patterns, are more likely to smoke, are less likely to have health insurance and usually have more physically and psychosocially demanding jobs [53]. These factors, combined with the recent physiological finding that lower socio-economic status is associated with higher levels of the stress hormones, cortisol and epinephrine [54], may explain the poorer HRQoL outcomes found with these patients.

Symptom status was also highly predictive of trajectory classification. The finding that higher NYHA dyspnoea class was predictive of *non-improver *membership for the PCS is also supported by previous studies [1]. Presumably patients who experience dyspnoea are more likely to have impaired left ventricular function and breathlessness, and are restricted in home, social or leisure activities [55] resulting in lower HRQoL.

It is also not surprising that lower scores on the POMS vigor-activity scale indicated *non-improver *membership of the PCS, and that higher scores on the POMS depresssion-dejection scale predicted *non-improver *membership for the MCS. These findings are consistent with two recent studies that have also found higher pre-operative depression scores to predict lower HRQoL six months after surgery [24,56]. It is known that depressed patients are less medically compliant [57], are less likely to exercise [58], and more likely to engage in unhealthy behaviours such as smoking [58], all factors which contribute to poorer HRQoL outcomes.

This study adds weight to the growing body of evidence that patients' perceived cognitive abilities may influence HRQoL outcomes. Lower scores on EFQ concentration were predictive of *non-improver *membership for the MCS. This finding is supported by a previous study which showed that perceived cognitive function, reflecting ability to concentrate, was a major determinant of HRQoL outcomes in a cross-sectional study of Swedish CABGS patients [59]. It has been suggested that poorer cognitive function impedes recovery, particularly in the context of CR, because it impacts on the patient's ability to learn or effectively respond to new information [60]. Further, it has been found that cognitive deficits after CABGS are associated with less ability to engage in activities of daily living, higher depression, more self-reported mental difficulties and greater symptom limitations [23] which result in poorer HRQoL outcomes.

The present study also improved upon previous studies by assessing the impact of operational and post-surgical variables. It has been argued that HRQoL in the months after CABGS may be affected by environmental factors such as processes and structures of care, complications of the surgery, or interim life change or health events [1,14]. The present study demonstrated that PCS group membership was adversely affected by surgical variables such as experiencing a new cardiac arrhythmia and the recording of higher pulmonary blood pressure during surgery. MCS group membership was not significantly associated with any of these variables in the final logistic regression. Attendance at CR was significantly associated with MCS *improver *group when medical variables were analysed separately, but was not significant in the presence of competing variables. This finding is consistent with other studies that have found little or no difference between CR attenders and non-attenders in HRQoL measured by the SF-36 [7,32]. It has been suggested that CR is not sufficiently intensive to influence recovery of HRQoL [7]. CR was only found to benefit physical function in a recent randomised controlled trial of an 18-session program which compared CR with usual care [61].

The main limitations of the present study concern the fact that it relied on postal collection of data with over half of the questionnaires not being returned. This limitation raises the possibility that the results may not be generalisable to all CABGS patients. Those who did not return the questionnaire had lower rates of high cholesterol and were less likely to have a positive family history of heart disease. However, in all other medical variables there were no significant differences between questionnaire returners and non-returners. Moreover, there were no differences with regard to gender and age. Other socio-demographic measures such as education level and employment status, were not investigated in excluded patients, so the possibility that non participants differed in these characteristics has not been examined.

The present study relied upon self-report for psychosocial measures and HRQoL rather than formal diagnostic criteria. Although the questionnaire order was varied, the possibility of a self-report bias exists. It has been shown that patients with depressive symptoms may over-report negative aspects of HRQoL and a spurious exposure-outcome association may be generated [62]. The inclusion of a self-report measure of cognitive functioning rather than an objective test battery may also be considered a limitation of the present study. However, it has been argued that neuropsychological test batteries that are often used in these studies may be too insensitive to measure small but personally, significant cognitive decline and that self-reported data may be of more value [59].

Another possible limitation of the study is the reported lack of responsiveness of the SF-36. Hawkes and colleagues argue that the SF-36 may not be sufficiently responsive as an outcome measure with CR patients [32]. Devon [63] compared the SF-36 with three other commonly used instruments, and found that it did not demonstrate responsiveness to change in functional status. Future studies might need to include a disease-specific measure of HRQoL, such as the MacNew HRQoL instrument [64], so that better responsiveness to change can be observed following a cardiac event such as CABGS.

## Conclusion

The findings of the present study highlight the probability that sub-groups of patients exist within the CABGS population, each with its own trajectory of HRQoL following surgery, which may not necessarily be linear. The results further imply that particular socio-demographic, medical and symptom-related variables are useful in predicting membership of these sub-groups. When considering these predictors of HRQoL outcomes, it is important to consider a comprehensive model that incorporates pre-operative, operative and post-operative variables, psychosocial and cognitive symptoms, and characteristics of the individual and the environment. Furthermore, development of interventions to improve HRQoL outcomes and enhanced clinical decision making may result from improved identification of predictors of HRQoL after CABGS.

## Abbreviations

CABGS Coronary artery bypass graft surgery

HRQoL Health related quality of life

NYHA New York Heart Association

GCM Growth curve modelling

GMM Growth mixture modelling

SF-36 The (Short-Form) SF-36

PCS Physical component summary

MCS Mental component summary

POMS Profile of Mood States

EFQ Functioning Questionnaire

BMI Body mass index

CR Cardiac rehabilitation

CFI Comparative Fit Index

BIC Bayesian Information Criterion

MI Myocardial infarction

## Competing interests

The author(s) declare that they have no competing interests.

## Authors' contributions

MLG drafted the manuscript and analysed the data using SPSS. PE supervised the data analysis, assisted with the interpretation of findings, and analysed the data using M-Plus. MW, BM and AJG participated in the design of the study. CE and RH participated in the coordination of the study. All authors read and approved the final manuscript.
